# The minimal important change for measures of balance and postural control in older adults: a systematic review

**DOI:** 10.1093/ageing/afac284

**Published:** 2022-12-19

**Authors:** Daniel C Low, Gregory S Walsh

**Affiliations:** Centre for Physical Activity in Health and Disease, Brunel University London, London, UK; Department of Sport, Health Sciences and Social Work, Oxford Brookes University, Oxford, UK

**Keywords:** minimum important change (MIC), responsiveness, minimal clinical important change (MCIC), older adults, systematic review, older people

## Abstract

The minimal important change and analogous terms (MIC) can provide a measure of change in health outcome variables that is associated with a level of importance for participant/patient. This review explores the availability of the MIC for different balance measures used with older adults in research and clinical settings. PubMed, ProQuest and Web of Science search engines were used and based on the inclusion and exclusion criteria, 11 studies were deemed suitable for data extraction and analysis. The results demonstrated that MIC is available for the following balance-associated tests: Berg Balance Scale, Timed Up and Go, Short Physical Performance Battery, BESTest and the Tinetti test. A range of MIC values were shown, reflective of different older adult health conditions, calculation methods and anchors used. It was also evident that the responsiveness of the test was not always available or appropriately determined, questioning the validity of the MIC value published. Greater research is needed to establish MIC for balance measurements for use with older adults with different health conditions, preferably using objective measures such as falls. The calculation of such statistics will improve the evaluation of intervention effectiveness.

## Key Points

- MIC values are available for some but not all balance measures used with older adults.
- A range of values and study heterogeneity means that if these values are to be used, caution is needed.
- Future research is needed to establish MIC values so that interventions are appropriately assessed.

## Introduction

Falls are a major problem for older adults, leading to negative physical, psychological, and social health and well-being, and premature death [1–3]. The term balance refers to the maintenance of the centre of mass (COM) over the base of support (BOS) [4] and can be applied to both static or dynamic movement. Falls commonly occur when this COM position cannot be maintained inside the BOS or controlled when it passes outside the BOS [5, 6], and muscular force is unable to act against gravity to keep the body in an upright standing position [4].

Changes due to aging can negatively impact balance and postural control strategies [7, 8], increasing the likelihood of a fall [9] and impairing the ability to perform everyday activities [10, 11]. This is related to changes to the musculoskeletal, neural and sensory systems (i.e. vestibular system, vision and proprioception) [12, 13], which play an interactive role in balance maintenance [14].

A plethora of measurements are available to assess balance and postural control, which clinicians use in the process of recognising and supporting rehabilitation needs of individuals. Researchers tend to use these tests to make generalisations regarding age-related changes or the effect of interventions on balance and postural control to a larger population. One major limitation of exploring change in this way is that it does not provide insight into the degree of importance that the change in an outcome variable represents for the individual participant/patient.

The term responsiveness describes whether a measurement can detect important changes in performance and is considered a measure of longitudinal validity [15, 16]. When responsiveness is assured, the minimal important change (MIC) can be used to recognise the minimum threshold for within-person change in an outcome variable that participants/patients would feel as important [17]. Similarly, the minimal clinical important change (MCIC) can be used to demonstrate the smallest change deemed sufficiently important from a clinical perspective [18]. These values are key for the evaluation of interventions and can also be used to plan sample sizes in trials [18, 19].

There are different methods for estimating the MIC/MCIC, which impact the calculated value [20, 21]. Similarly, the population on which the statistic is determined can affect the magnitude [17, 22], making using existing values with new populations problematic. Furthermore, there is inconsistency in terminology used in literature (e.g. MIC, minimal important difference, minimal clinically important difference, meaningful change threshold), which may make it hard to find an appropriate statistic to use.

For the purpose of this review, the term MIC will be used to encompass MIC and MCIC and all other analogous terms. Since a summary of MIC values for commonly used balance-related measures, separated by older adult health characteristics, is unavailable, the aim of this paper is to systematically review and summarise the literature reporting MIC for balance-related measurements, calculated on older adults with different health conditions. This review will offer clinicians and researchers clarity regarding which value to use, whilst also recognising where values are not available and thus where research is needed. Since the concept of responsiveness underpins the MIC statistic, the review will also provide a summary of the responsiveness statistics reported in these studies.

## Methods

### Search strategy and selection criteria

The study protocol for this systematic review was published on the PROSPERO Register of Systematic Reviews prior to the literature evaluation and data extraction (Prospero registration number: CRD42022309772). PubMed, ProQuest and Web of Science databases were searched using the terms presented in Table 1 for all years up to the 11 February 2022; a review of the reference lists of the eligible studies was also performed.

**Table 1:** Systematic search strategy

Search focus	Terms
Population	Elderly OR aging OR aging OR old OR older OR geriatric
Tests	balance OR ‘single-leg^*^ stan^*^’ OR ‘stand time’ OR ‘stance time’ OR ‘single leg stan^*^’ OR ‘single legged stan^*^’ OR ‘Berg Balance Scale’ OR ‘Tinetti balance assessment’ OR ‘timed up and go’ OR ‘functional reach’ OR ‘Romberg’ OR ‘Short Physical Performance Battery’ OR ‘limits of stability’ OR ‘centre of pressure’ OR ‘centre of pressure’ OR ‘sway’ OR ‘postural control’ OR ‘centre of mass’ OR ‘centre of mass’
Outcome	‘minimal important change’ OR ‘minimal important difference’ OR ‘minimal clinically important difference’ OR ‘minimal clinically important change’ OR ‘meaningful change threshold’ OR ‘minimal clinically important increase’ OR ‘minimal clinically important decrease’

To be included in this review, studies had to have a mean sample age of 60 years or greater, performed an assessment of balance, calculate the MIC using anchor- or vignette-based methods, with a longitudinal study design and be written in English. Studies were excluded if the MIC statistic were calculated using distribution-based methods since they measure change that is detectable rather than important [17]. The health status of participants/patients was not considered an exclusion criterion, but instead was used to compare MIC across sub-categories of older adult. The calculation of the MIC statistic could occur in studies that explore unintentional or natural change in balance or postural control, due for example, to injury or illness recovery; it could also occur intentionally following an intervention. No restriction on the intervention used was applied, as long as it had a within-subjects design; those studies which failed to meet the inclusion criteria were excluded.

The title and abstract of all records returned by the literature search were screened by both authors of this study independently against the inclusion criteria. Following title and abstract screening, the full text of remaining eligible records was retrieved and were reviewed by each author independently. At each stage of the screening, any discrepancies were resolved following discussion between the reviewers.

### Data extraction and synthesis

For those studies meeting the inclusion criteria, all MIC values were extracted. Additionally, COSMIN guidance [16, 23] was used to direct data extraction, ensuring important methodological features of responsiveness studies were highlighted (e.g. duration of longitudinal period, intervention details, inclusion of hypotheses, the anchors used and percentage of individuals that changed on the anchor). Likewise, criterion and construct responsiveness approach statistic(s), such as the area under curve (AUC) analysis and correlations between the outcome variable and anchor were extracted along with the calculation method. These were synthesised into tables with qualitative commentary. Additionally, data on the average age, sex percentage, sample size and health condition/status of the participant/patients were extracted to offer insight into the homogeneity between studies.

### Risk of bias

The two authors of this paper assessed the risk of bias (ROB) for each paper independently and then discussed conflicting reviews, coming to an agreement in all cases. The responsiveness ROB assessment tool used was described by COSMIN [23]. The overall ROB was assessed using ‘the worst score counts’ principle [23].

To assess responsiveness, the authors of this study needed to establish whether the research reported criterion or construct responsiveness approach. This is determined via the anchors used to assess change in a health outcome measure. When the anchor was considered the gold standard and the comparison is aimed at evaluating the predictive quality of the outcome variable in relation to this standard, criterion responsiveness approach was used [16]. A gold standard anchor can be defined as that which may not be the perfect test, but is the best available and has a standard with known results [24]; AUC is commonly used to assess this form of responsiveness [25]. On the other hand, a construct approach is used when gold standards are unknown or when the perception of global change in the body or health is of interest. This is known as the Therapist or Patient global rating of change (GRC), with patients and therapists often required to rate the perceived level of change in an outcome on a predetermined numerical scale. These anchors are based on subjective ratings and do not directly measure a problem [16]; the anchor may therefore not provide completely accurate estimates of the true health phenomenon [26]. Correlations and hypotheses are used when construct approach are reported [25]. Despite the differences, the anchors offer insight into the change in an individual condition from different perspectives. As such, this study will offer MIC data in context of their anchor measurement. Furthermore, regardless of whether the assessment used a construct or criterion approach, all responsiveness data for corresponding MIC values were reported in the main results.

## Results

### Search results

**Figure 1:**
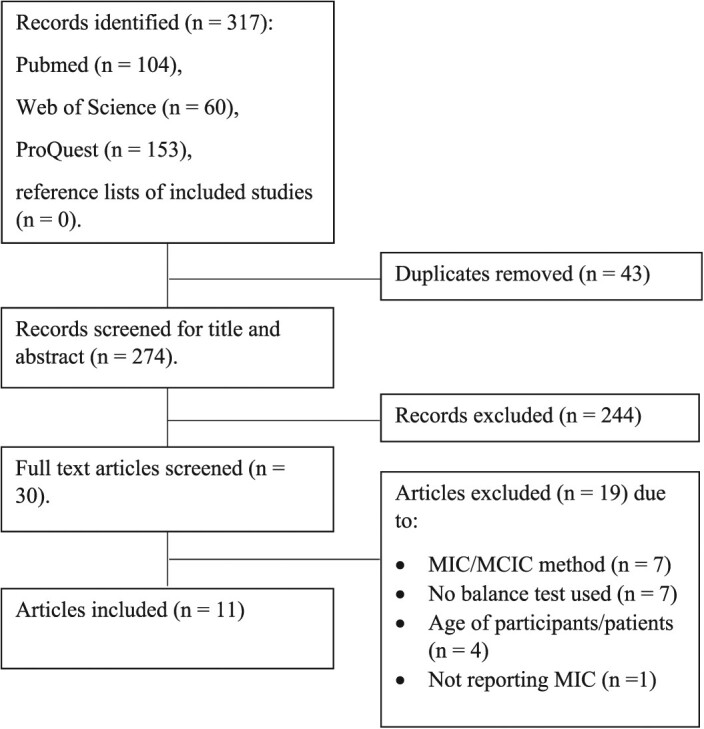
Flow of studies through the review process.

A total of 317 records were identified from the initial literature search, of which 43 were duplicate articles so were removed. Following the review of title and abstract and full text articles, a total of 11 studies met the inclusion criteria and were included in the review (Figure 1).

### Included study characteristics

All descriptive information is presented in the supplementary materials. MIC was calculated in the older adult populations who had Parkinson’s Disease [27], COPD [28, 29], neurological or neuromuscular disorders [30], stroke [31, 32], hip fracture [33] or total knee arthroplasty [34]. Older adults were also characterised as being post-acute cardiac patients [35], or had idiopathic normal pressure hydrocephalus [36], or were hospitalised with cognitive spectrum disorders [37]. There were differences in the proportion of males and females (ranging from 6.3 to 68% males) and average age of the older adult population (60.8 to 83.7 years of age).

Nine studies reported MIC before and after a physical therapy/rehabilitation or medical intervention [27–31, 33–36]. Two studies reported the MIC before and after a period of inpatient care without specifying an intervention [32, 37]. The follow up duration ranged from 2–4 hours to 17 weeks apart and the percentage of the population who changed on the anchor over this time was given in all but two studies [31, 33]. Given the combined differences between studies, there was considerable heterogeneity noted.

### MIC and responsiveness outcomes

Full details on the responsiveness of the outcome variables can be found in Table 2, and MIC information is presented in Table 3. Construct or criterion approach statistics were reported in all studies. In seven studies, construct approach could be identified via the reporting of Pearson’s, Spearman’s rank or Kendall’s tau-b correlations coefficient [27–32, 34, 37]. Braun et al. [37] demonstrated responsiveness (*r* > 0.3) for short physical performance battery (SPPB) when using the functional ambulation categories (FAC) and therapist GRC amount (T-GRC-A) anchors but not for the patient GRC amount (P-GRC-A) anchor; the authors also failed to show responsiveness for the timed up and go (TUG) for any anchor used. It was also demonstrated for Berg Balance Scale (BBS) in three studies, using Patient GRC (P-GRC), Patient/Therapist GRC (PT-GRC) and Patient Global Impression of Change (PGIC) anchors, respectively [28, 30, 32], although contrary findings were shown for the Functional Gait Assessment (FGA) anchor [34]. Finally, responsiveness was shown for the BESTest and modified versions using the P-GRC [27, 28], Therapist GRC (T-PGC) [27, 31], PT-GRC [30], FGA [34], six-minute walk test [29] and Modified British Medical Research Council dyspnoea scale (mMRC) [29] anchors.

**Table 2:** Responsiveness of the balance-related measures collected using older adults

	Berg Balance Scale		Timed Up and Go		Short Physical Performance Battery		Tineti		BESTest	
	Construct	Criterion	Construct	Criterion	Construct	Criterion	Construct	Criterion	Construct	Criterion
Godi [27]	–	–	–	–	–	–	–	–	P-GRC 0.42 T-GRC 0.62	P-GRC 0.75 T-GRC 0.82
Beauchamp [28]	P-GRC 0.5	P-GRC a little better 0.80 much better 0.74	–	–	–	–	–	–	P-GRC 0.5	P-GRC a little better 0.86 much better 0.76
Paixão [29]	–	–	–	–	–	–	–	–	mMRC −0.31 6-minute walk distance 0.37	mMRC 0.64 6-minute walk distance 0.63
Godi [30]	PT-GRC 0.62	PT-GRC 0.91	–	–	–	–	–	–	PT-GRC 0.72	PT-GRC 0.92
Beauchamp [31]	–	–	–	–	–	–	–	–	Mini-BESTest T-GRC 0.33	Mini-BESTest T-GRC 0.77
Saso [32]	PGIC −0.43 (All)	PGIC 0.78 (All) 0.77 (Mild stroke)	–	–	–	–	–	–	–	–
Tamura [33]	Not provided	FAC change 0.76 (All patients) 0.89 (assisted walking) 0.60 (unassisted walking) Substantial Change 0.81 (All patients) 0.80 (assisted walking) 0.81 (unassisted walking)	–	–	–	–	–	–	–	–
Chan [34]	FGA 0.15	FGA 0.59	–	–	–	–	–	–	FGA BESTest 0.55 Mini BESTest 0.52 Brief BESTest 0.40	FGA BESTest 0.81 Mini BESTest 0.78 Brief BESTest 0.71
Rinaldo [35]	–	–	–	–	PGIC Not provided	PGIC 0.77	–	–	–	–
Gallagher [36]	P-GRC (Balance) Not provided	P-GRC balance Moderate change 0.78 Significant change 0.74	P-GRC gait Not provided	P-GRC gait Moderate change 0.68 (TUG) 0.75 (TUG cognition) Significant change 0.71 (TUG) 0.75 (TUG cognition)	–	–	P-GRC balance Not provided GRC gait Not provided	P-GRC balance Moderate change 0.70 (Tinetti overall) 0.65 (Tinetti balance) 0.69 (Tinetti gait) Significant change 0.71 (Tinetti overall) 0.62 (Tinetti balance) 0.67 (Tinetti gait)	–	–
Braun [37]	–	–	P-GRC-A 0.19 T-GRC-A 0.17 FAC-C −0.12	P-GRC-A 0.55^*^ T-GRC-A 0.43^*^ FAC-C 0.57^*^	P-GRC-A 0.28 T-GRC-A 0.33 FAC-C 0.55	P-GRC-A 0.68^*^ T-GRC-A 0.69^*^ FAC-C 0.79^*^	–	–	–	–

Patient Global Rating of Change Amount (P-GRC-A); Therapist Global Rating of Change Amount (T-GRC-A); Functional Ambulation Categories (FAC); Patient Global Rating of Change (P-GRC); Global Rating of Change scale by patient and Therapist (mean score used) (PT-GRC); Patient Global Impression of Change (PGIC); Patient Global Rating of Change (P-GRC) balance and gait; Health Assessment Questionnaire Disability Index (HAQDI); Therapist Global Rating of Change (T-GRC); Modified British Medical Research Council dyspnoea scale (mMRC)

^^*^^AUC converted to decimal

**Table 3:** MIC of balance-related measures collected on older adults

	Berg Balance Scale	Timed Up and Go	Short Physical Performance Battery	Tineti	BESTest
Godi [27]	–	–	–	–	Mini-BESTest P-GRC 4 T-GRC 4
Beauchamp [28]	P-GRC a little better 3.5 points Much better 4.5 points Mean change a little better 4.8 points Much better 7.1 points	–	–	–	GRC a little better 10.2 points Much better 11.1 points Mean change a little better 12.6 points Much better 17.4 points
Paixão [29]	–	–	–	–	mMRC mean change 3.6 Regression 3.3 6-minute walk distance mean change 3.4 Regression 2.6
Godi [30]	PT-GRC Moderate improvement 6 points Mean change Null/small improvement 1.9 points Moderate improvement 7.0 points Large improvement 9.2 points	–	–	–	^*^mini-BESTest GRC Moderate improvement 4 points Mean change Null/small improvement 1.6 points Moderate improvement 4.6 points Large improvement 7.0 points
Beauchamp [31]	–	–	–	–	Mini-BESTest T-GRC 1
Saso [32]	PGIC 5.5 points (All) 5.5 points (Mild stroke)	–	–	–	–
Tamura [33]	FAC change 11.5 points (All patients) 10.5 points (assisted walking) 2.5 points (unassisted walking) Substantial change 18.5 points (All patients) 17.5 points (assisted walking) 24.5 points (unassisted walking)	–	–	–	–
Chan [34]	FGA 5 points	–	–	–	FGA BESTest 8 (out of 108 total score) Mini BESTest 2 (out of 28 total score) Brief BESTest 3 (out of 24 total score)
Rinaldo [35]	–	–	P-GRC 1 point	–	–
Gallagher [36]	P-GRC balance Moderate change 4 points Significant change 4 points	P-GRC gait Moderate change 3.63 sec (TUG) 2.60 sec (TUG cognition) Significant change 3.63 sec (TUG) 2.60 sec (TUG cognition)	–	P-GRC balance Moderate change 4 points (Tinitti overall) 2 points (Tinitti balance component) Significant change 4 points (Tinitti overall) 2 points (Tinitti balance component) P-GRC gait Moderate change 2 points (Tinitti gait component) Significant change 2 points (Tinitti gait component)	–
Braun [37]	–	P-GRC-I (*n* = 22) 2.0–3.4 seconds T-GRC-I (*n* = 22) 1.6–8.3 seconds FAC-C (*n* = 24) 1.8–2.0 seconds	P-GRC-I (*n* = 54) 0.5–1.5 points T-GRC-I (*n* = 54) 0.5–1.5 points FAC-C (*n* = 61) 0.5 points Mean change using T-GRC-I 0.4 points	–	–

Patient Global Rating of Change Amount (P-GRC-A); Therapist Global Rating of Change Amount (T-GRC-A); Functional Ambulation Categories (FAC); Patient Global Rating of Change (P-GRC); Global Rating of Change scale by patient and Therapist (mean score used) (PT-GRC); Patient Global Impression of Change (PGIC); Patient Global Rating of Change (P-GRC) balance and gait; Health Assessment Questionnaire Disability Index (HAQDI); Therapist Global Rating of Change (T-GRC); Modified British Medical Research Council dyspnea scale (mMRC)

Criterion approach to responsiveness indicated via AUC was satisfactory or greater (>0.7) for the SPPB test when using the PGIC [35] and FAC anchor [37] but not when using P-GRC-A or T-GRC-A anchors [37]. At least satisfactory responsiveness was shown for the BBS using P-GRC [28, 36], PT-GRC [30] and PGIC [32] anchors. Tamura et al. [33] also showed responsiveness for the BBS with a small change in FAC anchor when all participants and walking assisted participants were used but not when the population was categorised as unassisted walking; responsiveness was however shown for all groups when the change in anchor was considered ‘substantial’. Furthermore, Chan [34] failed to show responsiveness for the BBS using an FGA anchor. For the TUG, Braun et al. [37] failed to show responsiveness using P-GRC-A, T-GRC-A or FAC anchors and Gallagher et al. [36] showed responsiveness for the TUG cognition group, performing a counting task, but not the TUG only group using P-GRC anchor for a ‘moderate’ change in the anchor; both groups met the responsiveness criteria when change in anchor was ‘significant’. When using P-GRC with the full Tinetti test, Gallagher et al. [36] showed responsiveness (moderate and significant anchor change). However, when the balance or gait components were explored separately no responsiveness was shown. Finally, responsiveness was shown for the BESTest using P-GRC [27, 28], T-GRC [27, 31], PT-GRC [30] and FGA [34] but not six-minute walk test [29] or mMRC [29].

MIC was available for the following test measures: BBS [28, 30, 32–34, 36], Tinetti balance assessment [36], TUG [36, 37], SPPB [35, 37] and BESTest [27–31, 34]. MIC was calculated via ROC and AUC in all but one study, which reported MIC via the mean change method and through regression [29]; three other studies also reported additional MIC statistics, calculated via mean change method [28, 30, 37]. The range of values retrieved was 1.9 to 24.5 points (BBS), 0.4 to 1.5 points (SPPB), 1 to 17.4 (BESTest) and 1.6 to 8.3 s (TUG). For the Tinetti test, 2 points were reported for the Tinetti test balance and gait components only and 4 points for the overall test [36].

### Risk of bias

ROB assessment is given in Table 4. Across all studies, none of the anchors used were considered gold standard and thus N/A was considered appropriate to questions 1 to 3. All studies were therefore considered to explore the construct responsiveness approach and so questions 4 to 7 were answered for all studies. There were two studies where sub-group comparisons were made [32, 33]; thus, questions 8–10 were considered for these studies. Finally, nine studies used interventions [27–31, 33–36] and thus 11–13 were relevant to these studies.

**Table 4:** Risk of Bias Evaluation

	Risk of Bias Question									
Reference	For continuous scores: Were correlations between change scores, or the area under the ROC curve calculated?	For dichotomous scales: Were sensitivity and specificity (changed versus not changed) determined?	Is it clear what the comparator instrument(s) measure(s)?	Were the measurement properties of the comparator instrument(s) sufficient?	Were design and statistical methods adequate for the hypotheses to be tested?	Were there any other important flaws in the design or statistical methods of the study?	Was an adequate description provided of important characteristics of the subgroups?	Were design and statistical methods adequate for the hypotheses to be tested?	Was an adequate description provided of the intervention given?	Were design and statistical methods adequate for the hypotheses to be tested?
Godi 27	NA	NA	Very good	Very good	Very good	Very good	NA	NA	Very good	Very good
Beauchamp 28	NA	NA	Very good	Very good	Adequate	Very good	NA	NA	Very good	Adequate
Paixao 29	NA	NA	Very good	Very good	Very good	Very good	NA	NA	Very good	Very good
Godi 30	NA	NA	Very good	Very good	Adequate	Very good	NA	NA	Very good	Adequate
Beauchamp 31	NA	NA	Very good	Very good	Adequate	Doubtful	NA	NA	Very good	Adequate
Saso 32	NA	NA	Very good	Very good	Adequate	Very good	Very good	Adequate	NA	NA
Tamura 33	NA	NA	Very good	Very good	Doubtful	Doubtful	Very good	Doubtful	Very good	Doubtful
Chan 34	NA	NA	Very good	Very good	Adequate	Very good	NA	NA	Very good	Adequate
Rinaldo 35	NA	NA	Very good	Very good	Doubtful	Very good	NA	NA	Very good	Doubtful
Gallagher 36	NA	NA	Very good	Very good	Doubtful	Very good	NA	NA	Very good	Doubtful
Braun 37	NA	NA	Very good	Very good	Very good	Very good	NA	NA	NA	NA

The constructs were well described in all studies and was categorised as ‘very good’. The measurement properties of the anchor were also given ‘very good’ in all studies. Three studies reported study hypotheses [27, 29, 37], these were scored ‘very good’ regarding the appropriateness of statistical methods to test study hypotheses, since they all report correlation statistics. When hypotheses were not reported but where correlations between the anchor and outcome variable are reported, the appropriateness was deemed ‘adequate’ [28, 30–32, 34]. When studies failed to report correlations, and only report the AUC, these studies are deemed ‘doubtful’ in this regard [33, 35, 36]. Regarding the appropriateness of the methodological design, all studies were longitudinal in design and reported the length of time between repeated data collection; however, two studies failed to report the percentage of the sample that had changed over the longitudinal duration [31, 33], which was considered a minor methodological flaw; these studies were given a rating of ‘doubtful’. In the two studies where sub-group comparisons were made, the information provided regarding the group characteristics was rated as ‘very good’; this was also true for the intervention information given in those studies using interventions. The overall quality of the studies was scored as ‘very good’ in three studies [27, 29, 37], ‘adequate’ in four studies [28, 30, 32, 34] and ‘doubtful’ in four studies [31, 33, 35, 36].

## Discussion

The current study provides a novel systematic review of the MIC values available for balance-related measurements in older adults. The review reveals that values are available for BBS, Tinetti, TUG, SPPB and BESTtest, but not for measures of postural control or single leg standing, despite their use in literature evaluating older adults.

The MIC was most frequently reported for the BBS and BESTest; however, for both tests, there was a relatively large range in the reported MIC values (BBS: 3–44% and BESTest: 1–16% of the respective maximum possible score). Additionally, the range of values reported for SPPB was 3–12.5% of the total score. There was an even larger range of values for the TUG, which represented a change of 20–102% for adults aged 60–69 years and 13–69% for adults 86–89 years of age, estimated using literature reporting mean values [38, 39].

The MIC ranges found in this review provide insight for interpreting previous and subsequent intervention outcomes. For example, many interventions report significant changes in the balance tests cited and interpret these values to be an improvement in balance and physical function [40–45]. However, using the average change in scores for groups performing these tests and the standard deviation, score can fall outside of the MIC values range identified by the current review suggesting some or all participants failed to reach the MIC. For example, Spina et al. [44] demonstrated that following balance training, individuals with mild Parkinson’s Disease (PD) showed a 3.45-point difference for the Mini-BESTest, which was significant to *P* < 0.016. This difference is smaller than the 4-point MIC reported by Godi [27] for a similar population. On the other hand, using older adult COPD patients, Tounsi et al. [45] reported a significant change in BBS following an intervention of 4.6 points (*P* < 0.05); this is greater than the MIC reported by Beauchamp et al. [28] using a P-GRC anchor. In both cases, the standard deviation suggests that some but not all participants would fall within this range. It would have therefore been informative had the percentage of those which met the MIC been reported to fully appreciate the effectiveness of the intervention.

Guralnik et al. [46] suggests that meaningful change is context, perspective and purpose dependent. In agreement, the broad range of MIC values reflects differences in the health characteristic of the older adult population [22], as well as gender and age within each study. Furthermore, differences may occur due to the varied calculation method used to establish the MIC [17, 37]; this includes the method used to calculate optimal cut off and the choice between mean change or AUC analysis. There was also evidence that the anchor used can impact the MIC determined [37]. Similarly, there were differences in the approach to classify an important change (index of meaningfulness). Some authors report MIC using both small or large changes on the anchor [28, 30, 33, 36]. Furthermore, four studies report the use of a score of more than 2 on the GRC scale as important [31, 35–37], albeit with varying scales used (5, 6, 7 and 15 points), whereas two others use a score of more than 3 points on the GRC scale as important [27, 30]. This signifies poor clarity regarding the minimum change in the anchor that is deemed important. The anchors also often explored different but related concepts, asking questions about balance and mobility change, which will have likely impacted the MIC determined. The combined impact of this is that if this MIC is to be used by others in the critical evaluation of interventions and treatments [17] and in sample size calculations [18, 19], caution will be needed. Guralnik et al. [46] also suggested that measurements such as P-GRC are related to the beliefs and behaviours of the participants, making it hard to generalise the results across populations. To counter this, goal setting can be a useful when evaluating an intervention. Furthermore, no study explored negative change in balance measurement, which may be used to explore unintentional change due to disease or aging. The MIC is also missing for a range of older adult populations and tests and none of the anchors provided a direct measure of falls risk change; future research is needed in this area. Future studies should also consider whether the sample size used is justifiable since this information was only available in 6 of the 11 studies [28, 31, 34–37].

This review also provides a summary regarding the responsiveness of each balance-related measurement. Responsiveness was not demonstrated for all reported MIC values and thus it is not appropriate to use these MIC [22]. In some cases, this is pointed out by the authors [33, 34, 37]; however, this was not always the case [36]. Furthermore, based on the COSMIN guidelines [25], some studies report the criterion responsiveness approach inappropriately when using GRC or Patient Global Impression of Change, which are not considered gold standard anchors. Others report measures such as the Health Assessment Questionnaire and functional movement assessments that are subjective ratings; these studies fail to offer evidence as to why these should be considered gold standard measurements. Consequently, some may therefore consider these studies as failing to offer appropriate insight into the responsiveness of the data, questioning the usefulness of the MIC calculated. These concerns add a further consideration for those wishing to use these statistics in their evaluations. This review also highlighted that most studies possessed an adequate or doubtful ROB and thus future research needs to consider the appropriate design of responsiveness studies more closely.

An important point to highlight is that the MIC does not provide insight into other concepts that reflect meaningful change, such as sufficiently important difference [47] or smallest worthwhile effect [48–50]. Furthermore, it is acknowledged that as the estimated MIC value is derived from a wider sample of patients, the threshold may not apply for a specific patient [17]. Another limitation of this study was that the search criteria focused on balance measurements common within older adult research literature, yet other measures used in clinical settings may have been missed.

## Conclusion

This systematic review provides a summary of the MIC statistics for balance-related measurements. Given the range of MIC values and the heterogeneity of the populations, sampled clinicians and researchers should consider these factors and use caution when using the presented statistics to evaluate interventions. However, the values can be used as long as the impact of the responsiveness of the measurements and the methods/population used to determine the MIC is considered. Values are available for some, but not all balance-related tests or older-adult health condition, which suggests that future research is needed if participant/patient change is being appropriately assessed.

## Supplementary Material

aa-22-1228-File002_afac284
