# Supplementary material for: The minimal important change for measures of balance and postural control in older adults: a systematic review
Source: Age Ageing. 2022 Dec 19;51(12):afac284. doi: 10.1093/ageing/afac284 (PMC9799194; doi:10.1093/ageing/afac284)
Supplement: aa-22-1228-File002_afac284 [file aa-22-1228-file002_afac284.docx]

**The minimal important change for measures of balance and postural control in older adults: A systematic review**

Supplementary Table 1: Summary of the study method characteristics

| Author | Anchor | Total sample size (N) | Age (SD) | Male/ female percentage (%) | Health | Repeat duration | Construct Responsiveness: Hypothesis reported | Construct Responsiveness: Correlation between measure and anchor | Criterion Responsiveness: ROC and AUC | Minimal Important Change calculation method | Cause of change | Percent change in anchor |
| --- | --- | --- | --- | --- | --- | --- | --- | --- | --- | --- | --- | --- |
| Godi [27] | Patient Global Rating of (balance) Change (P-GRC)  Therapist Global Rating of (balance) Change (T-GRC) | 148 | 70.4 (8.4) | 55/45 | Mild to moderate Parkinson’s Disease | 4 weeks | No | Yes, Spearman’s rank | Yes | ROC/ AUC | Rehabilitation  treatment | P-GRC: 51% improved  T-GRC: 37% improved |
| Beauchamp  [28] | Patient Global Rating of (balance) Change (P-GRC) | 55 | 71.2 (7.1) | 44/56 | Chronic Obstructive Pulmonary disorder | 6 weeks | No | Yes, Spearman’s rank Correlation Coefficient | Yes | ROC/ AUC and Mean Change | Usual or balance-enhanced pulmonary rehabilitation training | 51%  much better  33% little better |
| Paixão [29] | Modified British Medical Research Council dyspnea scale (mMRC)  6-minute walk distance | 71 | 69 (8) | 76/24 | Chronic Obstructive Pulmonary disease | 12 weeks | No | Yes, Pearson’s Correlation Coefficient | Yes | Mean Change and Linear regression | Community-based Pulmonary Rehabilitation Programme | mMRC: 45% improved  6-MWD, 58% improved |
| Godi  [30] | Global Rating of (balance) Change using mean patient and therapist score (PT-GRC) | 93 | 66.2 (13.2) | 43/57 | Balance disorders | 2 weeks | No | Yes, Pearson’s Correlation Coefficient | Yes | ROC/ AUC and Mean Change | Physical therapy | 37% moderate improvement  6.4% large improvement |
| Beauchamp [31] | Therapist Global Rating of (balance) Change (T-GRC) | 50 | 60.8 (9.4) | 68/32 | Stroke | 17.4 ± 10.6 weeks | No | Yes, Spearman’s rank and Kendall’s tau-b | Yes | ROC/ AUC | Stroke rehabilitation | Not reported based on T-GRC anchor |
| Saso  [32] | Patient Global Impression of (balance) Change (PGIC) | 52  (All stroke)  32 (Mild stroke) | 78.7 (8.5) | 56/44 | Stroke | 1 month | No | Yes, Spearman’s rank Correlation coefficient | Yes | ROC/ AUC | Stroke in-patient unit | 35% responder |
| Tamura  [33] | Functional Ambulation Categories (FAC) | 187 | 83.7 (6.6) | 22/78 | Hip fracture | 51.3 ± 24.0 days | No | No | Yes | ROC/ AUC | Exercise rehabilitation programme | Unclear |
| Chan [34] | Functional Gait Assessment (FGA) | 134 | 66.3 (6.6) | 29/71 | Total Knee Arthroplasty | 2 weeks | No | Yes, Spearman’s rank Correlation, | Yes | ROC/ AUC | Outpatient physiotherapy | 54% improved |
| Rinaldo [35] | Patient Global Impression of (health status and physical performance) Change (PGIC) | 385 | 80.1 (3.7) | 58/42 | Post-acute cardiac surgery | 22.2 ± 10.4 days | No | No | Yes | ROC/ AUC | Cardiac rehabilitation | 92% improved |
| Gallagher [36] | Patient Global Rating of (balance and gait) Change (P-GRC) | 74 | 75 (IQR 68, 80) *median age | 64/36 | Idiopathic Normal Pressure Hydrocephalus | 2 to 4 hrs | No | No | Yes | ROC/ AUC | Tap test | P-GRC  Balance: 35% mild improvement  27% moderate improvement.  P-GRC  Gait: 28% reported moderate improvement  32% significant improvement. |
| Braun [37] | Patient Global Rating of (mobility) Change Amount (P-GRC-A)  Therapist Global Rating of (mobility) Change Amount (T-GRC-A)  Functional Ambulation Categories (FAC) | 63 (not all were included in calculations) | 83 (6) | 38/62 | Hospital patients with cognitive spectrum disorders | 7-21 days | Yes | Yes, Pearson’s and Spearman’s rank Correlation Coefficient | Yes | ROC/ AUC and Mean change | None – natural recovery change | P-GRC-A: 41% improved  T-GRC-A: 41%  improved  FAC: 32% improved |
